# Population prevalence of antiretroviral therapy sharing and its association with HIV viremia in rural Uganda: a cross‐sectional population‐based study

**DOI:** 10.1002/jia2.26135

**Published:** 2023-09-13

**Authors:** Caitlin E. Kennedy, Xinyi Feng, Robert Ssekubugu, Joseph G. Rosen, Joseph Ssekasanvu, Godfrey Kigozi, David Serwadda, Ping Teresa Yeh, Joseph Kagaayi, Thomas C. Quinn, Aaron A. R. Tobian, Maria J. Wawer, Steven J. Reynolds, Larry W. Chang, M. Kate Grabowski, Fred Nalugoda

**Affiliations:** ^1^ Social and Behavioral Interventions Program Department of International Health Johns Hopkins Bloomberg School of Public Health Baltimore Maryland USA; ^2^ Department of Pathology Johns Hopkins School of Medicine Baltimore Maryland USA; ^3^ Rakai Health Sciences Program Kalisizo Uganda; ^4^ Department of Epidemiology Johns Hopkins School of Medicine Baltimore Maryland USA; ^5^ Division of Intramural Research National Institute of Allergy and Infectious Diseases National Institutes of Health Bethesda Maryland USA; ^6^ Division of Infectious Diseases Department of Medicine Johns Hopkins School of Medicine Baltimore Maryland USA

**Keywords:** HIV care continuum, HIV adherence, ART sharing, ART division, viremia, Uganda

## Abstract

**Introduction:**

Antiretroviral treatment (ART) sharing has been reported among fishermen and sex workers in Uganda and South Africa. However, no population‐based studies have documented ART diversion prevalence (including sharing [giving/receiving], buying and selling) or its relationship with viremia among men and women living with HIV in Africa.

**Methods:**

In 2018–2020, we surveyed people living with HIV aged 15–49 years in 41 communities in the Rakai Community Cohort Study, a population‐based cohort in south‐central Uganda. We assessed the prevalence and correlates of self‐reported lifetime and past‐year ART diversion, stratifying by age and gender and documenting sources of diverted drugs. We used log‐binomial regression to quantify the relationship between diversion patterns and viremia (viral load >40 copies/ml), reported as unadjusted and adjusted prevalence ratios (aPR) with 95% confidence intervals (CI).

**Results:**

Of 2852 people living with HIV and self‐reporting current ART use, 266 (9.3%) reported lifetime ART diversion. Giving/receiving drugs were most common; few participants reported buying, and none reported selling. Men (12.9%) were more likely to report lifetime diversion than women (7.4%), with men aged 25–34 reporting high levels of sharing (18.9%). Friends were the most common sources of shared drugs, followed by spouses/sexual partners. Patterns of lifetime and past‐year diversion were similar. Among participants with viral load results, 8.6% were viraemic. In adjusted analyses, people who reported only giving ART were nearly twice as likely to be viraemic than those who reported no diversion (aPR: 1.94, 95% CI: 1.10−3.44), and those reporting only receiving ART were less likely to exhibit viremia (aPR: 0.46, 95% CI: 0.12−1.79), although the latter was not statistically significant. Reporting both giving and receiving ART was not associated with viremia (aPR: 0.79, 95% CI: 0.43−1.46). Reporting buying ART, though rare, was also correlated with higher rates of viremia, but this relationship was not statistically significant (aPR: 1.98, 95% CI: 0.72−5.45).

**Conclusions:**

ART sharing is common among persons reporting ART use in rural Uganda, particularly among men. Sharing ART was associated with viremia, and receiving ART may facilitate viral suppression. HIV programmes may benefit from considering ART sharing in counselling messages.

## 1 INTRODUCTION

Drug diversion is broadly conceptualized as the transfer of prescribed medication from the individual to whom it was prescribed to a different individual [1]. Drug diversion may include sharing (giving and receiving), buying or selling drugs. Drug sharing is frequently reported across classes of prescription medication, with rates of borrowing ranging from 5% to 52% across studies, and lending from 6% to 23% [2].

Globally, 38.4 million people are estimated to be living with HIV, 75% of whom are taking daily antiretroviral treatment (ART) [3]. Despite the enormous global attention to HIV treatment, there has been limited research on ART drug diversion. Research from the United States in South Florida has identified illicit marketplaces where ART is sold or traded [4, 5, 6, 7, 8, 9, 10, 11, 12]. Reported reasons for ART diversion in that setting include substance use, [4, 5, 8, 10], food and housing insecurity [6, 7, 8], barriers to accessing HIV care [4, 5, 8] and perceived efficacy of ART for HIV prevention (e.g. using ART for pre‐exposure prophylaxis [PrEP]) [10, 12]. Other studies from South Africa have reported ART use in a recreational drug cocktail [13, 14, 15, 16, 17]. These examples of drug diversion for illicit purposes were for ART combinations that contained efavirenz, and may be less likely to occur now following the switch to dolutegravir (DTG)‐based regimens.

While these studies are important, there have been few explorations of drug sharing for therapeutic HIV use, though drug sharing is common for other medications. To date, just one study has quantitatively assessed ART diversion for therapeutic use. This study among female sex workers living with HIV in eThekwini (Durban), South Africa, found that 30% reported ever sharing (giving and/or receiving) ART [18]. Factors associated with sharing ART included higher levels of alcohol use, illicit drug use, depression severity and physical/sexual violence [18], although in qualitative interviews, female sex workers reported they shared ART to avoid seeking clinical care or because they missed appointments [19]. The study found a modest dose−response relationship between the number of ART pills that participants reported giving to their peers in the last 30 days and viral suppression; however, there was no relationship between viral suppression and number of pills received or given [18].

In 2017, we conducted a qualitative study among female sex workers and fishermen living with HIV in hyperendemic fishing communities along Lake Victoria in Rakai, Uganda [20]. Participants reported frequent, episodic ART sharing with coworkers/friends, motivated by the desire to remain adherent to medication despite challenges to regular clinic attendance or stock‐outs. High HIV prevalence in these communities seemed to facilitate drug diversion, as participants were likely to know other people living with HIV. However, this qualitative study did not assess ART diversion among broader populations of people living with HIV, nor did it examine the relationship between diversion and health outcomes. Although our preliminary qualitative research found that ART diversion was used to support adherence and would thus be expected to be related to improved viral suppression, sharing drugs also implies insufficient ART access and running out of medications, so it may be associated with poorer adherence and reduced viral suppression.

Here, we sought to measure the prevalence of and factors associated with ART diversion, and to assess the relationship between ART diversion and viral suppression, in a representative population of people living with HIV in south‐central Uganda.

## 2 METHODS

### 2.1 Study setting and population

This study was conducted in Rakai and neighbouring districts in south‐central Uganda, where the Rakai Health Sciences Program (RHSP) has conducted epidemiological and HIV prevention research since 1988. RHSP conducts the Rakai Community Cohort Study (RCCS), an ongoing HIV surveillance study with over 30 years of population‐based, longitudinal data on HIV prevalence and incidence, risk behaviours, sexual networks, migration and service coverage [21]. Approximately every 18 months, the RCCS conducts household censuses, extensive interviews and HIV testing with all consenting individuals aged 15 and older in 41 study communities. These study communities are diverse and include inland agrarian villages, trading towns and fishing villages on Lake Victoria. Written informed consent is obtained from all adults and emancipated minors, and parental consent and minor assent is obtained for all unemancipated minors aged 15–17.

Questions about ART diversion were added to the RCCS survey round 19, conducted from 19 June 2018 to 6 November 2020. The analytic sample included all RCCS participants living with HIV aged 15–49 years who were aware of their HIV status and who self‐reported current ART use. The accuracy of self‐reported ART use in the cohort has previously been validated by plasma detection of antiretroviral drugs [22]. Associations with viral load were assessed among participants with viral load results.

### 2.2 Measures

To measure ART diversion, we asked participants to self‐report whether they had ever, or in the past 12 months, bought ART, sold ART, shared ART with someone else or had someone else share ART with them. For those who responded yes to any type of diversion, we elicited the drug recipients/sources involved in the reported diversion type (bought/sold/shared); responses included spouse/sexual partner, other family member, friend, work colleague, stranger or other.

HIV status was assessed using a validated three‐test algorithm followed by confirmatory enzyme immunoassays. Viral load assays were performed on stored plasma using the Abbott RealTime HIV‐1 assay (Abbott Molecular, Inc., Des Plaines, IL), and viremia was defined as having a detectable viral load (>40 copies/ml); we also ran sensitivity analyses with viral load ≥1000 copies/ml, the current World Health Organization (WHO) criteria [23].

Socio‐demographic measures included age, gender, marital status (never married, currently married, previously married [i.e. separated, divorced, widowed]), highest level of education (none, primary, secondary or higher), religion, occupation, migration status (recent in‐migrant to the community since the last survey round) and community of residence (fishing, trading or agrarian). Sexual behaviours in the past 12 months and other factors associated with HIV transmission were also assessed, including number and type of sexual partners (marital, non‐marital), condom use with non‐marital partners, exchange of money or goods for sex, alcohol use before sex (by self or partner), sex with partners outside their community and symptoms of genital ulcer disease. Current pregnancy status was assessed among women, and circumcision status was assessed among men.

### 2.3 Statistical analysis

We examined the prevalence of ART diversion overall among persons reporting ART use and by type of diversion, stratified by age, gender and community of residence, in the past 12 months and ever. We compared socio‐demographic characteristics and sexual behaviours among individuals who did and did not report ART diversion, and who reported different types of diversion using Pearson's Chi‐square test. We summarized data on drug recipients/sources by gender and type of ART diversion reported.

We used log‐binomial regression to quantify the relationship between lifetime ART diversion and viremia, reported as prevalence ratios (PR) and adjusted prevalence ratios (aPR) with 95% confidence intervals (CI). We hypothesized that individuals who reported giving or receiving ART would be more likely to be viraemic than those who did not report sharing. We first conducted unadjusted analyses for the whole sample, and stratified by gender and community of residence. We then conducted additional regression analyses adjusting for gender, age (in years) and community of residence (classified as fishing vs. non‐fishing [inland] communities).

## 3 RESULTS

Of 19,341 RCCS participants, 3374 (17%) were living with HIV, of whom 2852 (85%) self‐reported knowledge of their serostatus and currently taking ART. Of people living with HIV self‐reporting ART use, approximately two‐thirds were women (*n* = 1868/2852; 65%), and one‐third were men (*n* = 984/2852; 35%). Most were older than 35 years, had completed primary education or less and were employed in agricultural/housework, trading or fishing. Overall, 9.3% (*n* = 266/2852) reported lifetime ART diversion, while 6.8% (*n* = 193/2852) reported ART diversion in the past year.

**Figure 1:**
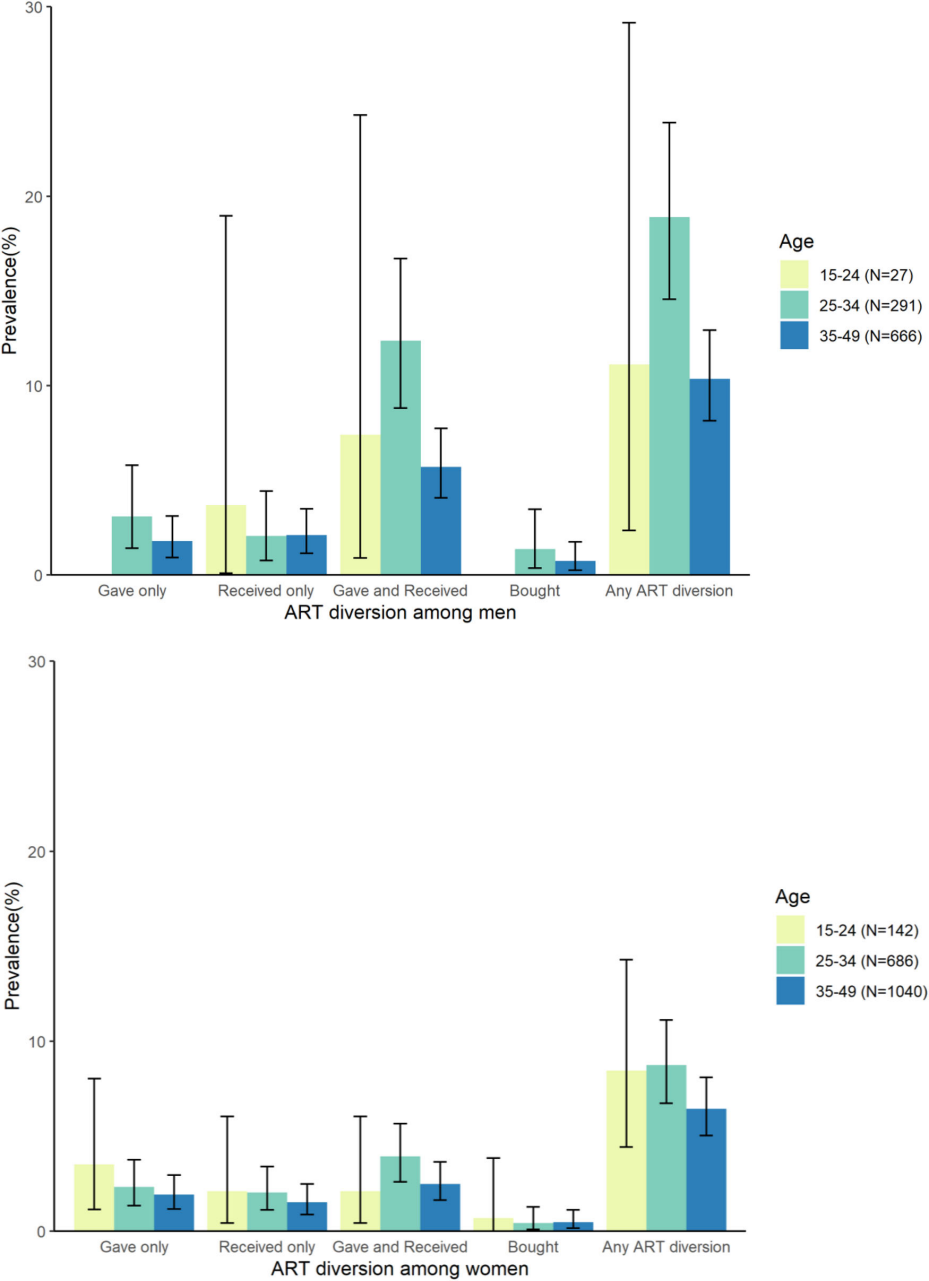
Prevalence of ever giving, receiving and buying antiretroviral treatment (ART) by age category and gender among people living with HIV ages 15–49 in 41 study communities in south‐central Uganda.

The practice of giving and receiving ART were the most commonly reported types of diversion; 62 participants (2.2%) reported giving ART only, 54 (1.9%) reported receiving ART only and 132 (4.6%) reported both giving and receiving (Table 1). Few participants reported buying ART (*n* = 18, 0.6%), and none reported selling. Men were more likely to report any diversion than women (12.9% vs. 7.4%). Stratifications by gender and age showed that men aged 25–34 years reported ART sharing most frequently (18.9%) (Figure 1). Patterns of giving, receiving, and buying ART and associations with socio‐demographic factors were similar for lifetime and past‐year diversion (Supplementary Table).

**Table 1:** Socio‐demographic factors associated with ever giving, receiving or buying ART among persons living with HIV self‐reporting ART use in south‐central Uganda (*N*=2852).

		ART diversion (*N*=266)					
Socio‐demographic factors	No ART diversion	Gave only	Received only	Gave and received	Bought	Any ART diversion	
	(*N*=2586)	*N*=62	*N*=54	*N*=132	*N*=18	*N*=266	*p*‐value*
Age (years)							
15−24	154 (91.1%)	5 (3.0%)	4 (2.4%)	5 (3.0%)	1 (0.6%)	15 (8.9%)	0.073
25−34	862 (88.2%)	25 (2.6%)	20 (2.1%)	63 (6.5%)	7 (0.7%)	115 (11.8%)	
35−49	1570 (92.0%)	32 (1.9%)	30 (1.8%)	64 (3.8%)	10 (0.6%)	136 (8.0%)	
Gender							
Female	1729 (92.6%)	41 (2.2%)	33 (1.8%)	56 (3.0%)	9 (0.5%)	139 (7.4%)	<0.001
Male	857 (87.1%)	21 (2.1%)	21 (2.1%)	76 (7.7%)	9 (0.9%)	127 (12.9%)	
Community of residence							
Agrarian	838 (94.9%)	9 (1.0%)	8 (0.9%)	24 (2.7%)	4 (0.5%)	45 (5.1%)	<0.001
Trading	582 (92.8%)	13 (2.1%)	13 (2.1%)	15 (2.4%)	4 (0.6%)	45 (7.2%)	
Fishing	1166 (86.9%)	40 (3.0%)	33 (2.5%)	93 (6.9%)	10 (0.8%)	176 (13.1%)	
Primary occupation							
Agriculture/housework	1008 (94.3%)	15 (1.4%)	10 (0.9%)	33 (3.1%)	3 (0.3%)	61 (5.7%)	<0.001
Bar/restaurant work	231 (87.8%)	9 (3.4%)	8 (3.0%)	12 (4.6%)	3 (1.1%)	32 (12.2%)	
Boda boda/trucking	22 (88.0%)	0 (0·0%)	2 (8.0%)	1 (4.0%)	0 (0.0%)	3 (12.0%)	
Fishing	338 (80.5%)	12 (2.9%)	10 (2.4%)	53 (12.6%)	7 (1.7%)	82 (19.5%)	
Student	5 (100.0%)	0 (0.0%)	0 (0.0%)	0 (0.0%)	0 (0.0%)	0 (0.0%)	
Trade/shop keeper	495 (92.4%)	10 (1.88%)	9 (1.7%)	18 (3.4%)	4 (0.8%)	41 (7.7%)	
Other	487 (91.2%)	16 (3.0%)	15 (2.8%)	15 (2.8%)	1 (0.2%)	47 (8.8%)	
Educational status							
None	479 (91.1%)	12 (2.3%)	11 (2.1%)	21 (4.0%)	3 (0.6%)	47 (8.9%)	0.240
Primary	1679 (90.2%)	41 (2.2%)	35 (1.9%)	98 (5.3%)	8 (0.4%)	182 (9.8%)	
Secondary	379 (91.6%)	8 (1.9%)	8 (1.9%)	13 (3.1%)	6 (1.5%)	35 (8.5%)	
Tertiary	49 (96.1%)	1 (2.0%)	0 (0.0%)	0 (0.0%)	1 (2.0%)	2 (3.9%)	
Religion							
Catholic	1731 (90.5%)	41 (2.1%)	33 (1.7%)	97 (5.1%)	11 (0.6%)	182 (9.5%)	0.088
Muslim	304 (90.8%)	8 (2.4%)	9 (2.7%)	13 (3.9%)	1 (0.3%)	31 (9.3%)	
Protestant	435 (91.8%)	11 (2.3%)	5 (1.1%)	17 (3.6%)	6 (1.3%)	39 (8.2%)	
Other	116 (89.2%)	2 (1.5%)	7 (5.4%)	5 (3.9%)	0 (0.0%)	14 (10.8%)	
Marital status							
Never married	149 (90.9%)	5 (3.1%)	4 (2.4%)	5 (3.1%)	1 (0.6%)	15 (9.2%)	0.230
Married, monogamous union	1237 (90.4%)	27 (2.0%)	25 (1.8%)	74 (5.4%)	6 (0.4%)	132 (9.6%)	
Married, polygamous union	321 (88.0%)	11 (3.0%)	6 (1.6%)	22 (6.0%)	5 (1.4%)	44 (12.1%)	
Previously married	879 (92.1%)	19 (2.0%)	19 (2.0%)	31 (3.3%)	6 (0.6%)	75 (7.9%)	
Migration status							
Recent in‐migrant	368 (91.5%)	7 (1.7%)	10 (2.5%)	14 (3.5%)	3 (0.8%)	34 (8.5%)	0.600
Pregnancy status (among women, *N*=1868)							
Pregnant	117 (90.0%)	3 (2.3%)	3 (2.3%)	5 (3.9%)	2 (1.5%)	13 (10.0%)	0.418

Note: No participants reported selling ART, so selling is not included in this table.

^*^ *p*‐value for chi‐square test by ART diversion types, comparing any antiretroviral treatment (ART) diversion (gave only, received only, gave and received, bought or sold) to no ART diversion. All *p*‐values were two‐sided and *p*<0.05 was considered statistically significant.

People who reported lifetime ART diversion were also generally more likely to report several behaviours associated with potential HIV transmission risk, including alcohol use before sex, sex with partners outside their community and symptoms of genital ulcer disease in the last 12 months (Table 2).

**Table 2:** Sexual behaviours associated with ever giving, receiving or buying ART among persons living with HIV self‐reporting ART use in south‐central Uganda (*N*=2852)

		ART diversion (*N*=266)					
	No ART diversion	Gave only	Received only	Gave and received	Bought	Any ART diversion	
Sexual behaviours	(*N*=2586)	*N*=62	*N*=54	*N*=132	*N*=18	*N*=266	*p*‐value*
Sexually active in the past year	2236 (90.2%)	56 (2.3%)	49 (2.0%)	122 (4.9%)	17 (0.7%)	244 (9.8%)	0.180
Multiple sexual partners in the past year	616 (85.4%)	23 (3.2%)	19 (2.6%)	55 (7.6%)	8 (1.1%)	105 (14.6%)	<0.001
Non‐marital partners in the past year (among married persons, *N*=2480)	980 (88.3%)	31 (2.8%)	26 (2.3%)	62 (5.6%)	11 (1.0%)	130 (11.7%)	0.055
Consistent condom use with non‐marital partners (among those with non‐marital partners, *N*=1110)	228 (89.1%)	5 (2.0%)	5 (2.0%)	13 (5.1%)	5 (2.0%)	28 (10.9%)	0.360
Alcohol use by respondents or partners before sex	1605 (88.3%)	50 (2.8%)	41 (2.3%)	106 (5.8%)	15 (0.8%)	212 (11.7%)	<0.001
Sex with partners outside community	174 (84.5%)	4 (1.9%)	6 (2.9%)	20 (9.7%)	2 (1.0%)	32 (15.5%)	0.013
Money, gifts or favours exchanged for sex with partner	294 (85.2%)	11 (3.2%)	13 (3.8%)	21 (6.1%)	6 (1.7%)	51 (14.8%)	0.052
Symptoms of genital ulcer disease in the last 12 months	130 (83.9%)	5 (3.2%)	7 (4.5%)	10 (6.5%)	3 (1.9%)	25 (16.1%)	0.010
Circumcised (among men, *N*=983)	461 (89.3%)	9 (1.7%)	11 (2.1%)	33 (6.4%)	2 (0.4%)	55 (10.7%)	0.495

Note: No participants reported selling ART, so selling is not included in this table.

^*^ *p*‐value for chi‐square test by ART diversion types, comparing any antiretroviral treatment (ART) diversion (gave only, received only, gave and received or bought) to no ART diversion. All *p*‐values were two‐sided and *p*<0.05 was considered statistically significant.

**Figure 2:**
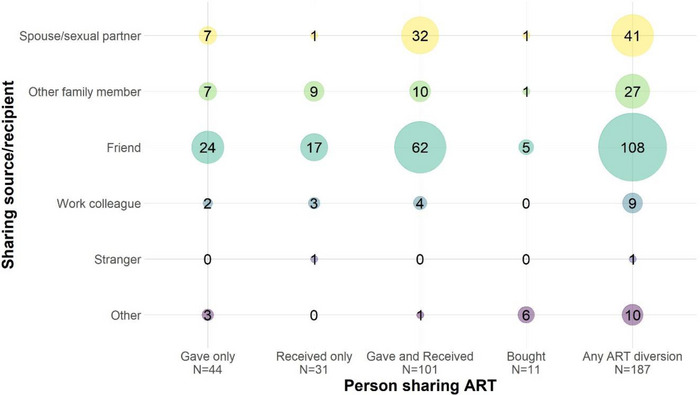
Recipients and sources of antiretroviral treatment (ART) given, received or bought in the past 12 months among persons living with HIV self‐reporting ART use and ART diversion in south‐central Uganda (*N*=187).

Friends were the most commonly reported recipients and sources of shared drugs: 57.8% (*n* = 108/187) of participants reporting any past‐year ART diversion said they had done so with a friend (Figure 2). Spouses/sexual partners were the next most common at 21.9% (*n* = 41/187), and other family members followed at 14.4% (*n* = 27/187). Almost no one reported sharing with strangers. Men and women generally reported similar recipients and sources of shared ART, although women (compared to men) were more likely to report sharing with other family members, while men (compared to women) were more likely to report sharing with work colleagues.

Of 2852 people living with HIV self‐reporting ART use, 2725 (95.5%) had viral load results, and 8.6% of these (234/2725) were viraemic. Associations between lifetime ART diversion and viremia were generally consistent in unadjusted, adjusted and stratified analyses. In adjusted analyses, people who reported only giving ART to others were nearly twice as likely to be viraemic than those who reported no diversion (aPR: 1.94, 95% CI: 1.10−3.44), while those who reported only receiving ART were less likely to be viraemic compared to those who reported no diversion, but this difference was not statistically significant (aPR: 0.46, 95% CI: 0.12−1.79) (Table 3). Reporting both giving and receiving was not associated with viremia (aPR: 0.79, 95% CI: 0.43−1.46). Reporting buying ART, though rare, also seemed associated with viremia, but was not statistically significant (aPR: 1.98, 95% CI: 0.72−5.45). Stratifications by gender yielded similar overall patterns but were not statistically significant. Sensitivity analyses with viral load ≥1000 copies/ml also yielded similar patterns, but only remained statistically significant for people reporting giving ART to others only.

**Table 3:** Regression results showing the association between sharing, giving and buying ART and viremia (viral load >40 copies/ml) among persons living with HIV self‐reporting ART use in south‐central Uganda (*N*=2725)

ART diversion	Viraemic/total (%)	PR^a^ (95% CI)	*p*‐value	aPR^b^ (95% CI)	*p*‐value
No diversion	209/2469 (8.5)	Ref	–	Ref	–
Gave only	10/58 (17.2)	2.04 (1.14−3.63)	0.016	1.94 (1.10, 3.44)	0.022
Received only	2/49 (4.1)	0.48 (0.12−1.89)	0.294	0.46 (0.12, 1.79)	0.261
Gave and received	10/131 (7.6)	0.90 (0.49−1.66)	0.740	0.79 (0.43, 1.46)	0.458
Bought	3/18 (16.7)	1.97 (0.70−5.58)	0.202	1.98 (0.72, 5.45)	0.185

Note: No participants reported selling ART, so selling is not included in this table.

^a^ Prevalence ratio (PR) and 95% confidence interval (CI), comparing the prevalence of viremia between a specific type of antiretroviral treatment (ART) diversion and no ART diversion.

^b^ Adjusted prevalence ratio (aPR), adjusting for sex, age in years and community type (fishing vs. non‐fishing).

## 4 DISCUSSION

To our knowledge, this is the first population‐based study documenting the prevalence of ART diversion and its relationship to HIV viremia among men and women living with HIV in the general population in Africa. We found that ART sharing is fairly common, particularly among men, and is associated with viremia.

We identified a relationship between ART sharing and viremia, with worse outcomes observed among people giving pills, potentially better outcomes observed among people receiving pills and no relationship seen among people both giving and receiving. These results suggest that sharing drugs with another person could precipitate a drug shortage for the lender (thus interfering with adherence and viral suppression), or people who are poorly adherent themselves may be more likely to share because they have extra pills available to give away. Conversely, borrowing drugs may facilitate treatment adherence for the borrower. Reporting both borrowing and sharing drugs may reflect a reciprocal arrangement to address more persistent barriers to ART access, which, on balance, may have no overall impact on adherence and viral suppression. Purchasing ART, although rare, may also be associated with viremia.

These findings differ from the single prior study among female sex workers in South Africa [18], which found that only giving was associated with higher (not lower) levels of viral suppression. That study used a different ART sharing measure and timeframe (number of pills shared in the last 30 days) and focused on sharing only with sex work colleagues and not sharing with other individuals, or buying and selling ART. It is thus unclear if the differing results are due to different methodological approaches or true underlying differences in different populations. Sex workers in South Africa may have strong relationships with each other due to the multiple stigmas and difficult life circumstances they face, which may not apply to our more diverse sample of men and women from rural communities in Uganda with a range of occupations and ART sharing partners. Ultimately, we feel these differences call for further research to better understand patterns of, reasons for and consequences of ART sharing across settings.

Men in our study were more likely to report sharing ART than women, and younger men (aged 25–34 years) had the highest overall levels of sharing. This group is also least likely to be virally suppressed in the RCCS overall. Our results are similar to findings from multiple countries in East and Southern Africa, where men are less likely to seek HIV testing, engage in care, adhere to ART or be virally suppressed [24]. Masculine norms likely influence these behaviours across settings [25], and health systems that are not aligned with men's health needs or social circumstances, such as work‐related mobility, may also prevent engagement in care [24]. We also found that ART sharing was associated with several sexual risk behaviours, suggesting potential gendered clustering of risk. Finally, we found that ART sharing occurred more frequently in fishing communities, which also have documented different patterns of sexual risk behaviours, migration, occupational experiences and social norms that may correlate with ART diversion [26, 27].

Friends were the most reported source or recipient of shared ART. This was somewhat surprising, since studies show other drugs are commonly shared among family members [2], and HIV often clusters within families. However, ART sharing with friends was consistent with findings from our previous qualitative work among fishermen and female sex workers [20]. Another study from northern Uganda also found that neighbours were the most reported source of shared prescription drugs, primarily pain medications [28]. Communal living situations and the high prevalence of HIV may make drug sharing more common among friends in our study setting. Reciprocity has also been described as “a core cultural value” [29] in Uganda, and social norms may support patterns of drug sharing among friends and neighbours in ways that differ from drug sharing in other settings.

The associations we found between ART sharing and viremia have programmatic implications. Although there has been a recent expansion of differentiated service delivery options that offer more flexible ART access, health system inflexibilities around ART distribution remain, and the lending/borrowing patterns we observed suggest continued ART access barriers. Currently, ART sharing is not discussed in Ugandan or WHO guidelines for ART counselling. Asking clients about their experiences with drug sharing, and discussing both the potential benefits and consequences of drug sharing, may improve counselling messages. ART sharing may also raise concerns around drug resistance. These concerns may be lessened as the majority of people on ART in Uganda are now prescribed ART regimens containing DTG, which are anticipated to have a higher genetic barrier to resistance [30], although there has been recent documentation of DTG resistance in African ART programmes [31].

Some research has suggested that antiretroviral drugs meant for ART [10, 12] or PrEP [32, 33] are shared with HIV‐negative individuals for HIV prevention, although other studies have shown PrEP sharing is rare [34]. We only included individuals who self‐reported knowing their HIV status and self‐reported being on ART, and we used the term ART rather than PrEP, which looks visibly different than ART pill formulations. We, therefore, think it was unlikely that we captured PrEP sharing. However, some proportion of participants who reported only giving ART to others may have been sharing with HIV‐negative individuals for prevention.

Our research was mostly conducted prior to the COVID‐19 pandemic in Uganda, during which ART distribution options became more flexible through expanded differentiated service delivery models, such as community client‐led ART delivery and community drug distribution points, and networks of ART sharing may have been disrupted due to COVID‐related mobility constraints during lockdown periods. However, RCCS surveys have found no change in viral suppression rates following COVID [35]. A study in Kampala, Uganda, found evidence suggesting that people living with HIV may have stockpiled ART tablets from previous prescriptions, which allowed them to keep taking their medication even when they could not visit ART clinics during COVID lockdowns [36]. We have heard similar anecdotes in clinical care that people may be getting ART from multiple clinics and giving or sharing with friends who feel too stigmatized to go themselves. Future research could examine whether clients get ART from multiple clinics to stockpile or share, and look at changes in ART diversion patterns and associations with service delivery models following COVID.

Strengths of our study include the large, population‐based sample for both men and women living with HIV across 41 study communities, and the availability of viral load biomarkers. Limitations include our reliance on self‐report for ART diversion measures, which may introduce recall or social desirability bias. In particular, the fact that a few participants reported buying ART but none reported selling ART may suggest the presence of social desirability bias. However, participants in our prior qualitative study were comfortable discussing ART giving and receiving with interviewers [20], and previous work in this setting has demonstrated the accuracy of other self‐reported ART‐related measures [22]. Further, the presence of social desirability bias likely means that our estimates of ART diversion are potential underestimates of the true extent of these behaviours. While the RCCS is a population‐based study, there may still be selection bias if non‐participants are more likely to be viraemic and also to share ART; however, the direction of such bias is unclear. We may not have accounted for all potential confounders in our adjusted analyses. Our cross‐sectional design cannot assess a causal relationship between ART diversion and viremia. Future longitudinal studies are needed to assess temporal relationships. Future studies could also examine associations between ART sharing and drug resistance.

## 5 CONCLUSIONS

In summary, this first population‐based assessment of ART diversion in Africa found that ART sharing is common, particularly among men. While receiving ART may support viral suppression, giving ART was associated with viremia. HIV programmes may benefit from considering drug sharing in counselling messaging.

## COMPETING INTERESTS

The authors have no competing interests to report.

## AUTHORS’ CONTRIBUTIONS

CEK, MKG, FN, SJR and LWC conceived and designed the study. In collaboration with MKG and CEK, JS and XF conducted statistical analysis and directly accessed and verified the underlying data reported in the manuscript. RS, GK and JK oversaw data collection. All authors had full access to the data in the study, participated in the interpretation of data and revising the manuscript, and had final responsibility for the decision to submit for publication.

## FUNDING

This study was funded by the U.S. National Institutes of Mental Health (NIMH) through grants R01MH105313 and R21AI145682 and in part by the Division of Intramural Research, National Institute of Allergy and Infectious Diseases. JGR was supported by a predoctoral training grant from NIMH (F31MH126796). The study sponsors had no role in the study design; in the collection, analysis or interpretation of data; in the writing of the report; or in the decision to submit the paper for publication.

## Supporting information


**Supplemental Table**. Sociodemographic factors and sexual behaviors associated with giving, receiving, or buying ART in the past 12 months among persons living with HIV self‐reporting ART use in south‐central Uganda (N = 2852)

## Data Availability

A de‐identified version of the Rakai Community Cohort Study data can be provided to interested parties subject to the completion of the Rakai Health Sciences Program data request form and signing of a Data Transfer Agreement. Inquiries should be directed to datarequests@rhsp.org.
