# Supplementary material for: Population prevalence of antiretroviral therapy sharing and its association with HIV viremia in rural Uganda: a cross‐sectional population‐based study
Source: J Int AIDS Soc. 2023 Sep 13;26(9):e26135. doi: 10.1002/jia2.26135 (PMC10500261; doi:10.1002/jia2.26135)
Supplement: Supplementary file 1 — Supplemental Table. Sociodemographic factors and sexual behaviors associated with giving, receiving, or buying ART in the past 12 months among persons living with HIV self‐reporting ART use in south‐central Uganda (N = 2852) [file JIA2-26-e26135-s001.docx]

**Supplemental Table. Sociodemographic factors and sexual behaviors associated with giving, receiving, or buying ART in the past 12 months among persons living with HIV self-reporting ART use in south-central Uganda (N=2852)**

| **Demographics** | **No ART diversion (N=2659)** | **ART diversion (N=193)** | | | | | |
| --- | --- | --- | --- | --- | --- | --- | --- |
|  |  | **Gave only** | **Received only** | **Gave and received** | **Bought** | **Any ART diversion**  **N=193** | **p-value** |
|  |  | **N=49** | **N=35** | **N=100** | **N=9** |  |  |
| **Age (years)** |  |  |  |  |  |  |  |
| 15-24 | 155 (91.7%) | 5 (3.0%) | 4 (2.4%) | 5 (3.0%) | 0 (0.0%) | 14 (8.3%) | 0·005 |
| 25-34 | 891 (91.2%) | 15 (1.5%) | 14 (1.4%) | 51 (5.2%) | 6 (0.6%) | 86 (8.8%) |  |
| 35-49 | 1613 (94.6%) | 29 (1.7%) | 17 (1.0%) | 44 (2.6%) | 3 (0.2%) | 93 (5.5%) |  |
| **Gender** |  |  |  |  |  |  |  |
| Female | 1780 (95.3%) | 28 (1.5%) | 22 (1.2%) | 33 (1.8%) | 5 (0.3%) | 88 (4.7%) | <0·001 |
| Male | 879 (89.3%) | 21 (2.1%) | 13 (1.3%) | 67 (6.8%) | 4 (0.4%) | 105 (10.7%) |  |
| **Community of residence** |  |  |  |  |  |  |  |
| Agrarian | 852 (96.5%) | 9 (1.0%) | 5 (0.6%) | 16 (1.8%) | 1 (0.1%) | 31 (3.5%) | <0·001 |
| Trading | 595 (94.9%) | 11 (1.8%) | 11 (1.8%) | 8 (1.3%) | 2 (0.3%) | 32 (5.1%) |  |
| Fishing | 1212 (90.3%) | 29 (2.2%) | 19 (1.4%) | 76 (5.7%) | 6 (0.5%) | 130 (9.7%) |  |
| **Primary occupation** |  |  |  |  |  |  |  |
| Agriculture/housework | 1023 (95.7%) | 14 (1.3%) | 6 (0.6%) | 24 (2.3%) | 2 (0.2%) | 46 (4.3%) | <0·001 |
| Bar/restaurant work | 246 (93.5%) | 4 (1.5%) | 4 (1.5%) | 7 (2.7%) | 2 (0.8%) | 17 (6.5%) |  |
| Boda boda/trucking | 22 (88.0%) | 0 (0.0%) | 2 (8.0%) | 1 (4.0%) | 0 (0.0%) | 3 (12.0%) |  |
| Fishing | 350 (83.3%) | 13 (3.1%) | 7 (1.7%) | 46 (11.0%) | 4 (1.0%) | 70 (16.7%) |  |
| Student | 5 (100%) | 0 (0.0%) | 0 (0.0%) | 0 (0.0%) | 0 (0.0%) | 0 (0.0%) |  |
| Trade/shop keeper | 512 (95.5%) | 7 (1.3%) | 7 (1.3%) | 9 (1.7%) | 1 (0.2%) | 24 (4.5%) |  |
| Other | 501 (93.8%) | 11 (2.1%) | 9 (1.7%) | 13 (2.4%) | 0 (0.0%) | 33 (6.2%) |  |
| **Educational status** |  |  |  |  |  |  |  |
| None | 493 (93.7%) | 10 (1.9%) | 6 (1.1%) | 17 (3.2%) | 0 (0.0%) | 33 (6.3%) | 0·750 |
| Primary | 1727 (92.8%) | 32 (1.7%) | 24 (1.3%) | 72 (3.9%) | 6 (0.3%) | 134 (7.2%) |  |
| Secondary | 390 (94.2%) | 6 (1.5%) | 4 (1.0%) | 11 (2.7%) | 3 (0.7%) | 24 (5.8%) |  |
| Tertiary | 49 (96.1%) | 1 (2.0%) | 1 (2.0%) | 0 (0.0%) | 0 (0.0%) | 2 (3.9%) |  |
| **Religion** |  |  |  |  |  |  |  |
| Catholic | 1769 (92.5%) | 34 (1.8%) | 23 (1.2%) | 81 (4.2%) | 6 (0.3%) | 144 (7.5%) | 0·017 |
| Muslim | 322 (96.1%) | 2 (0.6%) | 6 (1.8%) | 5 (1.5%) | 0 (0.0%) | 13 (3.9%) |  |
| Protestant | 446 (94.1%) | 12 (2.5%) | 2 (0.4%) | 11 (2.3%) | 3 (0.6%) | 28 (5.9%) |  |
| Other | 122 (93.9%) | 1 (0.8%) | 4 (3.1%) | 3 (2.3%) | 0 (0.0%) | 8 (6.1%) |  |
| **Marital status** |  |  |  |  |  |  |  |
| Never married | 153 (93.3%) | 2 (1.2%) | 3 (1.8%) | 5 (3.1%) | 1 (0.6%) | 11 (6.7%) | 0·026 |
| Married, monogamous union | 1269 (92.7%) | 20 (1.5%) | 17 (1.2%) | 60 (4.4%) | 3 (0.2%) | 100 (7.3%) |  |
| Married, polygamous union | 332 (91.0%) | 11 (3.0%) | 2 (0.6%) | 19 (5.2%) | 1 (0.3%) | 33 (9.0%) |  |
| Previously married | 905 (94.9%) | 16 (1.7%) | 13 (1.4%) | 16 (1.7%) | 4 (0.4%) | 49 (5.1%) |  |
| **Migration status** |  |  |  |  |  |  |  |
| Recent in-migrant | 374 (93.0%) | 5 (1.2%) | 9 (2.2%) | 12 (3.0%) | 2 (0.5%) | 28 (7.0%) | 0·250 |
| **Pregnancy status** (among women, N=1868) |  |  |  |  |  |  |  |
| Pregnant | 121 (93.1%) | 2 (1.5%) | 4 (3.1%) | 2 (1.5%) | 1 (0.8%) | 9 (6.9%) | 0·405 |
| **Sexual behaviors** |  |  |  |  |  |  |  |
| Sexually active in the past year | 2300 (92.7%) | 45 (1.8%) | 30 (1.2%) | 96 (3.9%) | 9 (0.4%) | 180 (7.3%) | 0·038 |
| Multiple sexual partners in the past year | 642 (89.0%) | 19 (2.6%) | 13 (1.8%) | 44 (6.1%) | 3 (0.4%) | 79 (11.0%) | <0·001 |
| Non-marital partners in the past year among married persons (N=2480) | 1014 (91.4%) | 25 (2.3%) | 17 (1.5%) | 48 (4.3%) | 6 (0.5%) | 96 (8.7%) | 0·130 |
| Consistent condom use with non-marital partners (N=1110) | 235 (91.8%) | 4 (1.6%) | 4 (1.6%) | 11 (4.3%) | 2 (0.8%) | 21 (8.2%) | 0·900 |
| Alcohol use by respondents or partners before sex | 1662 (91.5%) | 40 (2.2%) | 27 (1.5%) | 80 (4.4%) | 8 (0.4%) | 155 (8.5%) | <0·001 |
| Sex with partners outside community | 182 (88.4%) | 4 (1.9%) | 4 (1.9%) | 16 (7.8%) | 0 (0.0%) | 24 (11.7%) | 0·025 |
| Money, gifts, or favors exchanged for sex with partner | 305 (88.4%) | 9 (2.6%) | 9 (2.6%) | 18 (5.2%) | 4 (1.2%) | 40 (11.6%) | 0·027 |
| Symptoms of genital ulcer disease in the last 12 months | 138 (89.0%) | 4 (2.6%) | 6 (3.9%) | 7 (4.5%) | 0 (0.0%) | 17 (11.0%) | 0·023 |
| Circumcised (among men, N=983) | 471 (91.3%) | 10 (1.9%) | 7 (1.4%) | 28 (5.4%) | 0 (0.0%) | 45 (8.7%) | 0·405 |

^a^P-value for chi-square test by ART diversion types, comparing any antiretroviral treatment (ART) diversion (gave only, received only, gave and received, or bought) to no ART diversion. All p-values were two-sided and p<0.05 was considered statistically significant. Note: No participants reported selling ART, so selling is not included in this table.
